# Suicide Prevention Guideline Implementation in Specialist Mental Healthcare Institutions in The Netherlands

**DOI:** 10.3390/ijerph15050910

**Published:** 2018-05-03

**Authors:** Jan Mokkenstorm, Gerdien Franx, Renske Gilissen, Ad Kerkhof, Johannes Hendrikus Smit

**Affiliations:** 1 113 Suicide Prevention, 1105BP Amsterdam, The Netherlands; g.franx@113.nl (G.F.); r.gilissen@113.nl (R.G.); 2 Amsterdam Public Health, Department of Psychiatry, VU University Medical Center, 1081BT Amsterdam, The Netherlands; jh.smit@ggzingeest.nl; 3 Amsterdam Public Health, Department of Clinical Psychology, Faculty of Psychology and Education, VU University Amsterdam, 1081BT Amsterdam, The Netherlands; ajfm.kerkhof@vu.nl

**Keywords:** suicide prevention, implementation, practice guidelines, educational outreach, suicide, quality improvement, national strategy

## Abstract

In The Netherlands, on average 40% of all suicides concern patients treated by mental healthcare institutions (MHIs). Recent evidence indicates that implemented guideline recommendations significantly reduce the odds for patients to die by suicide. Implementation of the multidisciplinary guideline for diagnosis and treatment of suicidal behaviors is a main objective of the Dutch National Suicide Prevention Strategy. To this end, 24 MHIs that collectively reported 73% of patient suicides in 2015 received an educational outreach intervention offered by the national center of expertise. Aim: To investigate changes in levels of implementation of guideline recommendations; and to assess the degree of variation on suicide prevention policies and practices between MHIs. Methods: Implementation study with a prospective cohort design studying change over time on all domains of a Suicide Prevention Monitor, a guideline-based instrument assessing suicide prevention policies and practices within MHIs. Data were collected in six-month intervals between 2015 and 2017. Results: MHIs improved significantly on four out of ten domains: the development of an organizational suicide prevention policy; monitoring and trend-analysis of suicides numbers; evaluations after suicide; and clinician training. No improvement was measured on the domains pertaining to multi-annual training policies; collaborative care with external partners; recording and evaluation of suicide attempts; routine assessment of suicidality in all patients; safety planning and involving next of kin and carers. Furthermore, marked practice variation between MHIs was found which did not decrease over time. Conclusion: This study shows significant improvement in the implementation of four out of ten guideline-based suicide prevention policies in 24 specialist mental healthcare institutions in The Netherlands. The implementation level of suicide prevention policies and practices still appears to vary significantly between MHIs in The Netherlands.

## 1. Introduction

In The Netherlands, little progress has been made in structurally reducing suicide rates. Taking into account population growth and ageing, the Dutch suicide rate is at the level of the early 1990s (11.1 per 100 thousand residents). Between 2007 and 2013, the population number increased by 2% to 16.87 million and the annual number of suicides increased by 37% [1]. To address the problem of increased suicide rates in The Netherlands, the first National Suicide Prevention Strategy 2014–2017 was launched by the Dutch Ministry of Health, Welfare and Sports [2]. This Strategy was developed with over 20 stakeholders that committed to its realization. It contained 17 goals across 4 domains: healthcare, education, media and the social-economic sector. With the support of all stakeholders involved, 113 Suicide Prevention (from here on referred to as 113) was commissioned to promote and monitor the progress of the National Strategy including guideline implementation. Started in 2009 as an e-health platform for people at risk of suicide in 2009, 113 has developed into a national suicide prevention resource and expertise center. Apart from providing online anonymous healthcare, current activities include advocacy; creation of suicide prevention action networks with stakeholders; media surveillance; training and education; and consultancy on suicide prevention, quality improvement and implementation management. 

In mental healthcare, implementation of the Dutch multidisciplinary practice guideline for diagnosis and treatment of suicidal behavior (PGSB) [3] was chosen as a main objective of the Agenda. The PGSB was published in 2012 alongside with a Train-the-Trainer program that had been developed to support its dissemination and adoption [4]. 

Enhancing guideline implementation to reduce suicide rates has a clear rationale. On average, four out of ten suicides in The Netherlands concern patients treated by specialist mental healthcare institutions (MHIs) [5]. Recent evidence indicates that systematic and large-scale improvement of the quality and safety of healthcare services can significantly reduce suicide rates in clinical populations [6]. As Kapur et al. [7] showed, suicide rates dropped between 22% and 29% per implemented service improvement. 

Before publication of the PGSB, a marked degree of practice variation in the care for patients at risk of suicide in The Netherlands was observed [8]. Two out of three MHIs lacked well-defined suicide prevention standards. Guidelines that were used were often lacunar. Guideline implementation is intended to result in a higher quality of healthcare with lower practice variation across the country, eventually leading to lower suicide rates: it may take many years before implementation efforts take root on a scale large enough to improve the quality of care and to impact suicide rates [9].

Guideline implementation is notably hard, with barriers at the patient, professional, organizational and cultural level. Effective implementation starts by undertaking a situation analysis and selecting a strategy tailored to overcome present and expected barriers [10,11]. Important barriers to suicide prevention guideline implementation observed in Dutch MHIs were a lack of investment in quality improvement due to budget cuts and fear of blame. Although Dutch healthcare ranks among the best in the world [12], in 2006 a major healthcare reform was effectuated aimed at containing growing (mental) healthcare expenditure by a system of managed competition on healthcare markets. In 2012, mental healthcare budget cuts were intensified in response to the economic crisis and still-growing expenditure. In addition, MHIs experienced growing financial pressure by healthcare insurers to measure and report treatment outcomes [13]. 

Fear of blame was expressed during the development of the Strategy in 2013. Although they supported the general objectives of the Agenda, MHI representatives were concerned that unjustified expectations of guideline implementation would lead society to blame MHIs more often for not being able to prevent suicide. Commenting on the planned launch of the Agenda, the lead agency for Mental Healthcare providers (AMHAC-NL) stated that suicide rates are hard if not impossible to influence [14]. 

Given these barriers, it was apparent that guideline implementation and monitoring would require an approach that would optimally support, engage and involve MHIs, especially in the process of assessing the results of their achievements. To this end, 113 developed the Suicide Prevention by Educational Outreach protocol (SP-EDO). This multifaceted strategy combined two implementation strategies that emphasize improvement by learning: educational outreach and action research. The key component of this strategy was the Suicide Prevention Monitor instrument to guide and measure change in levels of implementation of 10 guideline-based suicide prevention recommendations. The primary aim of this study is to analyze change in the level of implementation of these policies in the course of a three-year period, from 2015 to 2017. The secondary aim is to assess the degree of practice variation between participant MHIs in the course of this period. These outcomes will be discussed and future developments will be explored. 

## 2. Methods

### 2.1. Design and Setting 

An observational study was conducted, assessing change in levels of implementation of guideline-based suicide prevention policies in specialist mental healthcare in The Netherlands between 2015 and 2017. This three-year time frame coincided with the launch of the first National Strategy 2014–2017. The study setting was specialist mental healthcare. Specialist mental healthcare is organized around 35 large MHIs in geographical catchment areas serving patients with severe and complex psychiatric and substance abuse disorders and acute psychosocial crisis. Typical MHI services include crisis resolution care; assertive outreach and recovery-oriented care; outpatient, day and inpatient psychiatric care; residential care; addiction and forensic care; general hospital consultation and liaison care. These services are offered to adults aged 18 and older including the elderly and can be accessed on general practitioner referral. Apart from waiting lists for some specialist treatments, access to care is generally good. Dutch inhabitants have mandatory insurance against the cost of healthcare with a fixed deductible for specialist care (currently €385 per year). For those with a low-income, insurance costs and deductibles can be alleviated by healthcare allowances and social assistance benefits. 

### 2.2. Participants

Participants were MHIs providing integrated specialist mental healthcare. Aiming for national coverage and representation with a limited time frame and budget, it was intended to include a cohort of MHIs that cumulatively report 70% of the patient suicides in specialist mental healthcare. Selection was based on the Dutch Healthcare Inspectorate listing of 182 MHIs that reported suicides between 2007 and 2012, of which 35 MHIs provide integral specialist mental healthcare [5]. This listing shows that the majority of MHIs report low numbers of suicides; and shows that a stable majority of 25 MHIs that are known to treat great numbers of patients report 10 or more suicides per year. Twenty five MHIs that reported 10 or more suicides in 2011 and 2012 were approached to participate, receiving an invitation letter with a follow-up telephone call to the MHI Board of Directors. One MHI declined due to a lack of time and different priorities. At the start of the study in 2015, the participant MHIs cumulatively reported 73% of the patient suicide mortality to the Dutch Healthcare Inspectorate (500 suicide deaths in the participant MHIs: 684 in all MHIs) with suicide rates ranging between 40 and 226 suicides per 100,000 patients, with an average of 113 suicides per 100,000. On average, the 24 included MHIs treated 21,500 patients per year.

All participant MHIs are governed by a board of directors that manage divisions and teams across the spectrum of specialist mental healthcare services. By law, a psychiatrist working as a non-board Chief Medical Officer (CMO) independently monitors the quality of care for patients. Within each MHI, a contact person was assigned to represent the organization and provide the information needed. Contact persons were either the CMO or a commissioned policy advisor supervised by the CMO or Board of Directors. 

### 2.3. The SP-EDO Implementation Strategy

SP-EDO was developed as a multifaceted implementation strategy, consisting of four components: change-agents, a suicide prevention monitor, feedback and dialogue sessions and meetings for learning and exchange. SP-EDO blends techniques derived from educational outreach or academic detailing described by Soumerai and Avorn [15] with principles of action research [16]. Educational outreach, or “academic detailing” is a frequently used implementation strategy which demonstrated moderate effects on improvement of patient care [10]. It is based on social influence theories of implementation, involving different techniques to increase knowledge, raise awareness and readiness for change, and provide positive reinforcement of improved practices. 

Action research recognizes that the process of observation of implementation by itself may positively or negatively influence the process of implementation. In the sensitive area of suicide prevention, measurement of levels of implementation may evoke defensive reporting or outright withdrawal to avoid the risk of being blamed or shamed. Thus, a design that strongly relies on objective measurement by an external agent in order to yield the hardest possible data may seriously backfire. Action research is an interactive inquiry process that balances problem-solving actions implemented in a collaborative context with short supportive cycles of data-driven research of the results of the implementation. Thus, data gathered for scientific purposes is used to drive learning and implementation cycles. 

In the present study, data were collected using a collaboratively developed Suicide Prevention Monitor and a scoring procedure based on consensus between the MHIs and change-agents. This collaborative approach was chosen to mitigate the risk of defensive MHI reporting or withdrawal that was to be expected given the ambivalence of MHIs towards suicide prevention guideline implementation and monitoring. While these consensus-based data may be regarded as less objective than external observations per se, they were considered valid enough to provide MHIs with a frame of reference to guide their actions and to scientifically assess the progress of implementation over time. 

#### 2.3.1. Change-Agents

**Figure 1:**
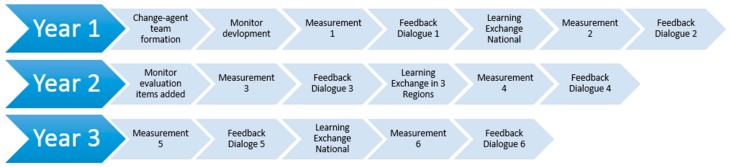
SP-EDO Study timeline summary of interventions and measurements.

To operationalize SP-EDO, a team of four change-agents was recruited based on their expertise in suicide prevention, marketing and detailing, and quality improvement. The team consisted of two senior consultants with extensive experience in the field of guideline development and implementation (Master level sociologist with a background in psychiatric nursing; Postdoc with a Master’s degree in Health Sciences and a background in nursing); a 113-psychologist/suicide prevention trainer; and a licensed psychosocial counselor with 10-year working experience as a pharmaceutical sales representative and sales manager. Two psychiatrists who provided guidance with respect to substantive mental healthcare issues supported the team. This team was sent out to establish a trusting relationship with MHI representatives in order to (1) develop and score the Suicide Prevention Monitor; (2) have dialogue and feedback sessions on MHIs’ progress of implementation; and (3) offer them learning and exchange meetings. Figure 1 presents a timeline overview of the SP-EDO implementation strategy. 

#### 2.3.2. Development of the Suicide Prevention Monitor

Instruments like toolkits with checklists, or self-study surveys have been recently developed to promote and guide suicide implementation efforts within mental healthcare [17,18]. Inspired by these examples, a Suicide Prevention Monitor was developed in collaboration with the participating MHIs in three stages. 

In the first stage, 113 proposed a prototype that was formatted as a list of policy recommendations on six domains. These six domains were chosen on the level of organizational policy: the general MHI suicide prevention policy; the monitoring and analysis of suicide numbers; learning and care improvement following suicide evaluation; collaborative care arrangements; multi-annual staff training policy and the percentage of clinicians trained to apply suicide prevention guideline recommendations. To stimulate self-reflection and to avoid eliciting defensive responses no predefined criteria for levels of implementation were provided to five pilot MHIs. MHIs were requested to self-rate their levels of implementation on a 1–5 visual analogue scale ranging from no implementation (red zone) to full implementation (green zone). Although this prototype offered a maximum of self-rating flexibility, none of five piloting MHIs proved able to self-rate their level of suicide prevention implementation on any of the domains. MHI representatives indicated that they lacked a frame of reference and invited 113 to add predefined levels of implementation to the Monitor. 

In the second stage of development, 113 proposed standard criteria defining levels of implementation on a 1–5 scale. Levels were defined to allow for most MHIs not to score red while keeping ample room for improvement to achieve perfection in suicide prevention and patient safety policies. This six-domain Suicide Prevention Monitor was used during the first two measurements. Notable MHI comments and attitudes towards the Monitor were summarized in a written report at the end of this period. Overall, the Monitor was positively received as a learning tool to support priority setting and provide incentives for improvement.

In the third stage, further development was based on critical feedback about definitions perceived to be unclear in some domains and 6-month measurement periods being too brief to capture change. This feedback resulted in two minor textual alterations to increase clarity in the level of implementation in the domain of training efforts. Apart from these minor alterations, the six domains and the definitions of levels of implementation remained unchanged. In addition, MHIs suggested that the Monitor focus more directly on suicide prevention practices in daily care. Following this suggestion, 4 additional domains with definitions of levels of implementation were added. These more practice-oriented domains included: recording of suicide attempts in electronic health records; percentage of patients assessed for suicidality in the course of treatment; involvement of family or carers of suicidal patients; safety planning and continuity of care. 

#### 2.3.3. Feedback and Dialogue Sessions

To raise the awareness and commitment and to build trusting relationships, 113 change-agents engaged MHIs in feedback and dialogue sessions. They contacted the MHIs every sixth months, with at least one face-to-face visit per year, to reflect on the implementation progress, the Monitor results provided by the organizations, on possible causes for lower scores and on suggestions for improvement activities. In the first year of the program two face-to-face sessions were planned, one at the MHI top leadership (CEO or Board of Directors) level, and the second at the practice level with clinicians of ‘suicide prevention teams’, multidisciplinary teams responsible for evaluating suicides and related policies within the organizations. In the second and third year, one face-to-face visit and one telephone contact was organized. Before each of the face-to-face sessions, a topic list was developed and shared with the organizations. Topics discussed in the very first meeting with MHI CEOs focused on contextual aspects such as the organization’s vision and policies concerning suicide prevention, participation with consumers and carers, and collaboration with partner organizations to guarantee continuity of care for suicidal patients. Topics discussed in subsequent contacts focused on the improvement on each domain of the monitor and on barriers and facilitators for making progress. 

#### 2.3.4. Learning and Exchange Meetings for MHI Professionals

During the three-year program, three regional and two national learning and exchange meetings for professionals were organized. Experts presented on selected subjects such as continuity of care and the perspectives of consumers or carers. 113 change agents used visually appealing graphical representations of monitor scores as input for discussion on the progress of the implementation and the degree of perceived policy variation. Opportunities were offered to exchange practices and practical ways to overcome barriers and to successfully implement recommendations for guidelines. These positive examples were recorded in professionally produced video clips published on the 113 website.

### 2.4. Outcome Measurement: The Suicide Prevention Monitor

Table 1 lists the domains of the Suicide Prevention Monitor with definitions of levels of implementation. 

### 2.5. Data Collection and Scoring Prcedure

Every six months, 113 change-agents contacted the contact person (CMO or a commissioned policy advisor supervised by the CMO) of each MHI. Rating of level of implementation was based on consensus between MHI contact persons and two 113 change-agents. Contacts were asked to assess implementation levels as defined in the Monitor tool and to substantiate their assessment with supporting documents. These documents included annual quality and safety reports on the organizational level and on the level of MHI suicide prevention taskforces; policies and reports on training and workforce development; demonstration of electronic health record facilities; signed agreements with healthcare network partners. 113 change-agents rated Monitor domains independently of each other and proposed a consensus rating to the MHI contact person. Scoring issues were regularly discussed in the change-agent team.

The Suicide Prevention Monitor scores were collected twice per year during three years, resulting in a total of six measurements for each participating MHI. During the first year, the Suicide Prevention Monitor consisted of six domains. In the last two years, four domains were added resulting in a ten-domain Suicide Prevention Monitor. 

### 2.6. Analysis

To detect changes in the level of implementation on each of the Monitor domains over time, a one-way repeated measures ANOVA was conducted with time (measurement 1/2/3/4/5/6) as within-subjects factor. The associations of Mauchly’s Test of Sphericity indicated that the assumption of sphericity had been violated (*p* < 0.001), and therefore, a Greenhouse–Geisser correction was used. To investigate if the practice variation between MHIs decreased over time, standard deviations were calculated for each monitor domain to quantify the amount of practice variation between MHIs and the degree of practice variation between MHIs during the first measurement was compared with the practice variation at measurement 6, using a paired samples *t*-test. 

## 3. Results

### 3.1. Implementation of Guideline-Informed Suicide Prevention Policies

Table 2 shows the overall progress in suicide prevention practices on each of the Monitor domains. Overall, in three years’ time, significant progress has been made F(3.5, 73.4) = 14.6, *p* < 0.001. Monitor scores increased on the domains “suicide prevention policy” (F(2.8, 55.9) = 11.6, *p* < 0.001), “monitoring of suicides” (F(3.0, 60.2) = 18.8, *p* < 0.001), “evaluation after suicide” (F(3.3, 65.4) = 3.1, *p* = 0.028) and “training of staff” (F(2.4, 47) = 12.3, *p* < 0.001). Pairwise comparisons using the Bonferroni correction show that improvement was significant in the first till third measurement on the domain “suicide prevention policy” (*p* = 0.005). On the domain ‘monitoring suicide numbers’ a significant improvement was shown from the first till second measurement (*p* < 0.001), and on the domain ‘suicide prevention training’ between measurement one and four (*p* = 0.033). After three assessments, no significant improvements were observed (Table 2).

### 3.2. Practice Variation

As reported by the one-way repeated measures ANOVA, between-institution effects were significant on each Monitor domain (*p* < 0.001), demonstrating reported practice variation between MHIs. There was no significant decline in practice variation between measurement one and six (t(5) = 2.4, *p* > 0.05). The overall progress from the first till sixth measurement varied between institutions from −0.3 (slight deterioration) to 2.1 (improvement). Institutions with lower scores at the first measurement improved more than institutions with higher first measurement scores (Pearson correlation −0.47, *p* < 0.05).

## 4. Discussion

Twenty-four MHIs received an educational outreach approach to improve the quality of their suicide prevention practices. A central element of the approach was the six-monthly rating on the Suicide Prevention Monitor. The observation that pilot MHIs were initially unable to self-rate their levels of implementation illustrates the width of the science-policy/practice gap at the beginning of the intervention. In the course of three years, MHIs improved on four out of ten domains of the Suicide Prevention Monitor: the development of an organizational suicide prevention policy; monitoring and trend-analysis of suicides numbers; evaluations after suicide; and clinician training. With respect to the latter: an impressive training effort has been made. Starting with on average less than 10%, at the end of three years MHIs report that 40–80% of all clinicians have been trained. The non-significant trend towards mandatory entrance and booster training in multi-annual training policies further indicates that MHIs recognize the need for workforce development in order to provide guideline-based suicide prevention care. Improvement in collaborative care with external partners was not significant, with MHIs on average improving between measurements 1–4 but leveling off in the last year. No improvement was measured on the domains pertaining to recording and evaluation of suicide attempts; routine assessment of suicidality in all patients; safety planning and involvement of relatives and carers. 

As the analyses indicate, there is significant variation in the quality of service policies and practices between MHIs. Considering that MHIs generally provide the same kind of services to the same categories of patients, this suggests that in The Netherlands similar patients at risk of suicide may receive different qualities of suicide prevention care, dependent on the providing MHI. In the course of the intervention period, MHIs with lower baseline scores improved faster than MHIs with higher baseline scores. However, MHI mean Monitor score variance was unchanged at measurement 6. Chances of patients to be asked about suicidality in the course of treatment, or to be treated by a clinician trained in guideline recommendations varied between 10% and 80%. This could imply that in The Netherlands still many patients at risk of suicide are not identified, are not offered a safety planning intervention, and receive no specific interventions to attenuate their suicidal behaviors. 

As trusting working relations developed, change-agents observed MHI contact persons to become growingly self-critical, with an increased sense of ownership of the need for implementation. This may in part explain the finding that the rate of improvement leveled off in time. Another explanation may be that, during the study period, professionals in many MHIs had to function under substantial pressure from increased regulatory and production requirements that were imposed by financers. In the course of the study, the balance in the trade-off between production goals and patient safety goals may have shifted back to production [19]. A final explanation of the rate of improvement leveling off in the course of time may be derived from the action research literature. This suggests a “valley” phase of hard work (“uphill battle”) that precedes a “victory phase” in which goals will be attained that were collaboratively developed in the high-energy initial “vision phase” [20]. 

As the change-agents reported in their feedback reports, the Suicide Prevention Monitor was generally positively received. The 10 Monitor domains became areas priority setting for local quality improvement activities in all participant MHIs. The opportunity to compare Monitor scores between MHIs aroused curiosity about the performance of other organizations. During exchange meetings, differences between MHI Monitor scores were explored in an atmosphere of learning and mutual support. Towards the end of the program, the instrument was used with an increased sense of ownership and accountability. It is plausible that typical elements of educational outreach as described by Soumerai and Avorn [15] have contributed to this positive attitudinal shift towards improving the care in order to prevent more suicides. These elements include establishing credibility through a respected organizational identity, referencing authoritative and unbiased sources of information, presenting both sides of controversial issues, stimulating active participation in educational interactions, using concise graphic educational materials, highlighting and repeating the essential messages, and providing positive reinforcement of improved practices in follow-up visits. In addition, the National Strategy as a whole evoked and received positive media attention. In six regions Suicide Prevention Action Network communities were formed, using the European Alliance Against Depression (EAAD) strategy that includes improving treatment of depression and suicidality in general practice, building chains of care with MHIs and a media campaign to reduce stigma and to stimulate help-seeking [21,22]. Finally, a number of regions and MHIs declared publicly to pursue a Zero Suicide ambition. These developments may have contributed to raised awareness and an increased sense of urgency and responsibility in MHIs. 

Our findings of six out of ten recommendations not improving in three years, and practice variation still marked across the country, are in line with previous research. It may take years to improve the quality of care for patients at risk of suicide. However, considering the devastating impact of suicide there is little time to lose. Rational interventions like training, education and monitoring are likely insufficient to bring about the change in culture and mindset needed to significantly improve the safety and the quality of care for suicidal patients on a national scale, within a decade. More is needed, starting with the establishment of a safer and just culture of improvement and learning [23] that allows for the pursuit of an ambitious, inspirational goal that clearly expresses that suicide is not an acceptable outcome of healthcare [24,25].

The results of this study have to be interpreted against the backdrop of some limitations of this research. This study did not objectively assess levels of implementation by investigating actual daily practice, but assessed this based on consensus rating by MHIs and 113 change-agents on domains that are likely to be of influence to daily practice. Because of its design lacking a control condition, it is not possible to make causal inferences between the educational outreach efforts and the implementation levels in MHIs. 

An important limitation is due to the nature of action research, with the observer and the observed interacting around the measurement on a domain they both have interests in. Although action research enables measurement of suicide prevention guideline implementation that would otherwise be hard, if not impossible, this methodology may entail a risk of observer bias. With suicide being a sensitive topic, this may have skewed MHI self-rating towards more positive scores. Still, not all domains improved and there was no improvement on the domains that MHIs themselves had suggested to monitor.

At this early stage of implementation, the Monitor focuses on policies and practices that were considered to create basic conditions for MHI suicide prevention care quality improvement. A limitation of this approach is that the Monitor does not cover all PGSB guideline recommendations; nor important evidence-based policies that are recommended from a Zero Suicide perspective [25,26,27], or that can be derived from recent research (e.g., the UK National Inquiry into Suicide and Homicide [18] and a meta-analysis of treatment modalities [28]). The Monitor did not assess the implementation policies regarding the use of suicide-specific treatment modalities; evidence-based treatment of depression; availability of addiction care; the removal of ligature points; absconding and no-show; timelines for the assessment of suicidality and for safety planning. Future editions of the Monitor should assess these quality indicators. To this end, a Suicide Prevention Team Monitor is now being piloted that assesses these quality indicators on the level of care delivered by MHI teams.

To our knowledge this paper is the first to report on the impact of systematic, national efforts to promote suicide prevention guideline implementation in the policies of MHIs. An important strength of this study is that it reports on the assessment of guideline implementation in a very sensitive area that may affect the quality of care for over 500,000 patients with severe mental illness. Collaborative development and scoring of the Monitor proved to be acceptable to MHIs and feasible for 113. Within the methodological constraints of self-rating and action research, the Monitor outcomes provided important insights to guide further efforts. A final important finding is that it is feasible to engage MHIs nationwide in a constructive dialogue about their suicide prevention policies and practices. Once started, no MHI withdrew. This finding may be relevant to consider for current and future national suicide prevention strategies. 

## 5. Future Developments

The National Suicide Prevention Strategy being continued, SP-EDO will continue with change-agents visiting MHIs and collaboratively scoring the Monitor for the next three years. New educational materials like e-learning and info-graphics will be developed to support learning and exchange meetings. Recently, 14 MHIs have joined forces to take quality and safety improvement to a next level. Together they formed the Suicide Prevention Action Network in Healthcare (SUPRANET Care) [29] with a Zero Suicide mission statement: to provide healthcare so good that no patient dies alone and in despair by suicide. In this confidential network, patient and practice data relevant for suicide prevention are shared and analyzed for meaningful trends. This allows for data-driven learning and improvement based on benchmark comparison and the exchange of good clinical practices and implementation strategies. SUPRANET Care will be supported by 113 change-agents and the SP-EDO project lead. As a result of this development, sector organization AMHAC-NL expressed great interest in promoting suicide prevention among its members, stimulating them to participate in SUPRANET Care and planning to spearhead suicide prevention in their strategy. 

## 6. Conclusions

During the National Suicide Prevention Strategy 2014–2017, the level of implementation of four guideline-informed suicide prevention policies in 24 specialist mental healthcare institutions in The Netherlands improved significantly. Six other suicide prevention policies and practices did not significantly improve. With regard to suicide prevention policies and practices, there is still marked variation between MHIs in the country. 

## Figures and Tables

**Table 1:** Suicide Prevention Monitor.

Domains Measurement 1–6	Level of Implementation
1. Suicide prevention policy on organizational level	No actual suicide prevention policy Policy < 5 years; contains 0–2 guideline recommendations Ibid 2 & >3 guideline recommendations Ibid 3 & reflects patients’ perspective Ibid 4 & part of general patient safety policy; clear prevention ambition
2. Monitoring suicide numbers	Incomplete monitoring of suicides, no analysis of trends Complete monitoring, no analysis of trends Complete monitoring, with analysis of trends & recommendations to improve Ibid 3 & Improvement plan across service after sharing w. patient advocacy Ibid 4 & transparent publication of trends, recommendations and plans
3. Evaluation & improvement after suicide	<75% of suicides are evaluated in a multidisciplinary team >75% evaluated by a multidisciplinary team using a guideline-based method or instrument Ibid 2 & Significant Others involved & requested to identify issues to improve Ibid 3 & at least 1 improvement plan completed & shared across service Ibid 4 & >25% of evaluations is based on extensive root cause analysis
4. Collaborative care	No written collaborative care agreements with regional partners Written agreements with partners, describing responsibilities Ibid 2 & including at least 2 healthcare partners (e.g., General Practitioners, Emergency Departments, Addiction Care) Ibid 3 & including at least 2 partners other than healthcare (e.g., police) Ibid 4 & including annual evaluation & update of agreement.
5. Multi-annual workforce training plan	There is no plan or a plan in development Plan is in development but not in effect. Complete, multi-annual plan leading to a competent present workforce Ibid 3 & Compulsory training for new employees Ibid 4 & Compulsory booster training for all employees
6. Suicide prevention training of clinicians	<1% of clinicians trained in the last 2 years 1–10% of clinicians trained in the last 2 years 11–40% of clinicians trained in the last 2 years 41–80% of clinicians trained in the last 2 years 81–100% of clinicians trained in the last 2 years
**Added Domains Measurement 3–6**	**Level of Implementation**
7. Recording of suicide attempts in Electronic Health Record (EHR)	<20% of known attempts are recorded 21–50% of known attempts are recorded 51–80% of known attempts 81–99% of known attempts recorded in a dedicated Electronic Health Record field or by Alert Ibid 4 & All attempts with serious medical consequences are evaluated
8. Assessment of suicidality	<20% of all patients assessed in course of treatment 20–50% of all patients assessed in course of treatment 51–80% assessed using systematic interview & reported in Electronic Health Record Ibid 3 & Alert in Electronic Health Record Ibid 4 100% of patients
9. Involving family/carers	<20% of suicidal patients has family/carers registered & involved at 1st contact Ibid 1 20–50% Ibid 2 51–80% & agreement on active involvement Ibid 3 81–99% Ibid 4 100% and actual involvement during entire treatment trajectory
10. Safety planning & Continuity of Care	Suicidal patients have no safety plan; continuity of care is not guaranteed Suicidal patients have a safety plan; continuity of care is not guaranteed Suicidal patient have a safety plan & guaranteed continuity & warm handoffs Ibid 3: safety plan has prominent place in Electronic Health Record Ibid 4: carers are actively involved in safety & continuity

**Table 2:** Mean rating on the Suicide Prevention Monitor in 24 Mental Healthcare organizations, measurement 1–6 (January 2015–June 2017).

Measurement	1 Mean (SD)	2 Mean (SD)	3 Mean (SD)	4 Mean (SD)	5 Mean (SD)	6 Mean (SD)	F
Total mean	2.8 (0.5)	3.3 (0.7)	3.2 (0.6)	3.3 (0.6)	3.4 (0.7)	3.5 (0.6)	14.6 ***
1. Suicide prevention policy	2.2 (1.2)	3.0 (1.3)	3.3 (1.2)	3.3 (1.2)	3.5 (1.2)	3.8 (0.9)	11.6 ***
2. Monitoring suicide numbers	2.3 (0.6)	3.3 (0.6)	3.3 (0.6)	3.3 (0.6)	3.4 (0.6)	3.4 (0.6)	18.8 ***
3. Evaluation/improvement after suicide	2.9 (1.1)	3.3 (1.1)	3.2 (1.4)	3.4 (1.4)	3.5 (1.4)	3.7 (1.1)	3.1 *
4. Collaborative care external partners	2.9 (1.2)	3.2 (1.2)	3.2 (1.3)	3.6 (1.2)	3.6 (1.2)	3.5 (1.1)	2.1
5. Multi-annual workforce training plan	3.3 (1.1)	3.4 (1.0)	3.5 (1.1)	3.5 (1.1)	3.7 (1.0)	3.8 (1.0)	2.7
6. Suicide prevention training	2.7 (1.2)	3.0 (1.2)	3.6 (1.1)	3.7 (1.1)	3.9 (1.1)	4.0 (0.9)	12.3 ***
7. Recording of suicide attempts in EHR			2.9 (1.0)	3.0 (1.0)	3.1 (0.9)	3.1 (1.0)	0.7
8. Assessment of suicidality			2.9 (0.9)	2.9 (0.9)	3.2 (1.1)	3.2 (0.9)	2.1
9. Involving family/carers			3.2 (0.9)	3.2 (1.0)	3.4 (0.9)	3.4 (0.9)	2.0
10. Safety planning & continuity of care			3.0 (1.1)	3.1 (1.1)	3.3 (1.0)	3.1 (1.0)	1.5

*** *p* < 0.001, * *p* < 0.05, higher Means imply better implementation of guidelines
